# Protocol, and practical challenges, for a randomised controlled trial comparing the impact of high intensity interval training against standard care before major abdominal surgery: study protocol for a randomised controlled trial

**DOI:** 10.1186/s13063-018-2701-9

**Published:** 2018-06-25

**Authors:** John Woodfield, Matthew Zacharias, Genevieve Wilson, Fran Munro, Kate Thomas, Andrew Gray, James Baldi

**Affiliations:** 1 Department of Surgery, University of Otago, Dunedin, New Zealand; 2 Department of Surgical Sciences, University of Otago, Dunedin, New Zealand; 3 Department of Anaesthesia, University of Otago, Dunedin, New Zealand; 4 Department of Medicine, University of Otago, Dunedin, New Zealand; 5 Department of Preventive and Social Medicine, University of Otago, Dunedin, New Zealand

**Keywords:** Prehabilitation, High intensity interval training, Postoperative complications, Cardiopulmonary exercise testing

## Abstract

**Background:**

Risk factors, such as the number of pre-existing co-morbidities, the extent of the underlying pathology and the magnitude of the required operation, cannot be changed before surgery. It may, however, be possible to improve the cardiopulmonary fitness of the patient with an individualised exercise program. We are performing a randomised controlled trial (RCT) assessing the impact of High Intensity Interval Training (HIIT) on preoperative cardiopulmonary fitness and postoperative outcomes in patients undergoing major abdominal surgery.

**Methods:**

Consecutive eligible patients undergoing elective abdominal surgery are being randomised to HIIT or standard care in a 1:1 ratio. Participants allocated to HIIT will perform 14 exercise sessions on a stationary cycle ergometer, over a period of 4–6 weeks before surgery. The sessions, which are individualised, aim to start with ten repeated 1-min blocks of intense exercise with a target of reaching a heart rate exceeding 90% of the age predicted maximum, followed by 1 min of lower intensity cycling. As endurance improves, the duration of exercise is increased to achieve five 2-min intervals of high intensity exercise followed by 2 min of lower intensity cycling. Each training session lasts approximately 30 min. The primary endpoint, change in peak oxygen consumption (Peak VO_2_) measured during cardiopulmonary exercise testing, is assessed at baseline and before surgery. Secondary endpoints include postoperative complications, length of hospital stay and three clinically validated scores: the surgical recovery scale; the postoperative morbidity survey; and the SF-36 quality of life score. The standard deviation for changes in Peak VO_2_ will be assessed after the first 30 patients and will be used to calculate the required sample size.

**Discussion:**

We want to assess if 14 sessions of HIIT is sufficient to improve Peak VO_2_ by 2 mL/kg/min in patients undergoing major abdominal surgery and to explore the best clinical endpoint for a subsequent RCT designed to assess if improving Peak VO_2_ will translate into improving clinical outcomes after surgery.

**Trial registration:**

Australian New Zealand Clinical Trials Registry, ACTRN12617000587303. Registered on 26 April 2017.

## Background

While complications continue to be a common event after abdominal surgery [1], the ability to predict who will develop perioperative complications remains difficult because the aetiology of adverse events is multifactorial [2]. In terms of reducing the likelihood of complications, many of the most important risk factors—such as the number of pre-existing co-morbidities, the extent of the underlying pathology and the magnitude of the required operation—cannot be changed before surgery. Stopping smoking [3] and optimising specific medical problems will improve the outcome in some patients. However, one important risk factor which it may be possible to improve in a significant group of patients is the functional reserve or the cardiopulmonary fitness of the patient.

Cardiopulmonary fitness can be assessed by measuring oxygen consumption during cardiopulmonary exercise testing (CPET). We know that this is an important clinical measurement as a poor oxygen consumption, including an anaerobic threshold (AT) of < 10-11 mL/kg/min [4–6] or a peak oxygen consumption (Peak VO_2_) of < 15–18.6 mL/kg/min [7], has been demonstrated to be associated with significantly higher rates of postoperative complications. In general terms, a low oxygen consumption is also predictive of a higher medium-term mortality risk [8], and it has been estimated that approximately half of patients presenting for intra-abdominal surgery do not have the prerequisite fitness on objective exercise testing to be considered at low risk for postoperative complications [9].

The main types of aerobic exercise which may improve oxygen consumption are High Intensity Interval Training (HIIT) and continuous moderate exercise. General studies (not perioperative) comparing the impact of these different types of exercise have shown that HIIT results in a more substantial [10] and faster [11, 12] improvement in Peak VO_2_ than continuous moderate exercise. The faster improvement is important in the context of surgery, as the urgency of the required surgery may mean that there is a limited period of time available between the time of diagnosis and surgery. There is also some evidence that a supervised HIIT program is safe in patients with moderate coronary artery disease and cardiac failure [12].

Although the general benefits of exercise have been widely studied and there is an increasing literature on the benefits of preoperative exercise, there are a number of important questions that need to be answered. Studies in the literature can be classified into four groups: (1) those that look at multimodal prehabilitation interventions including physical exercises (which may include muscle strengthening, aerobic and respiratory exercises), nutritional supplementation and psychological support; (2) those that assess aerobic exercises but do not measure oxygen consumption; (3) those that assess the impact of aerobic exercises on oxygen consumption; and (4) those that assess at the impact of HIIT on oxygen consumption. A review of prehabilitation [13] assessing the role of muscle strengthening, respiratory and aerobic exercises in orthopaedic, cardiac and abdominal surgery highlighted the potential of two weeks of inspiratory muscle training to reduce respiratory complications and to potentially reduce length of stay after cardiac and abdominal surgery. A recent review of prehabilitation, which identified nine studies using aerobic exercises before abdominal cancer surgery [14], consistently showed some improvements in the distance walked over 6 min or in oxygen consumption. However, only two of four studies found evidence that the quality of life was improved, only one of three found evidence that patient anxiety was decreased, and there was no identified reduction in postoperative complications. The main conclusion of this review was that the significant heterogeneity in almost all aspects of the identified studies made it difficult to come to any firm conclusions. They emphasised the need for greater standardisation in future studies.

We have identified ten studies that document the impact of preoperative aerobic exercise on either the anaerobic threshold or Peak VO_2_ in the 4–6 weeks before surgery [15–24]. All studies were on patients undergoing major abdominal or thoracic procedures. All except one [22] demonstrated an improvement in oxygen consumption. Two recent studies have prospectively assessed the impact of HIIT. In 2015, West et al. [15], in a non-randomised study which included 22 patients having 18 sessions of HIIT over six weeks after long course chemoradiotherapy for rectal cancer, demonstrated a 2.65 mL/kg/min recovery of maximal oxygen consumption back to the pre-chemoradiotherapy levels of oxygen consumption. In 2016, Dunne et al. [18], in a randomised study which included 19 patients having 12 sessions of HIIT over four weeks before liver surgery, demonstrated a 2.0 mL/kg/min improvement in Peak VO_2_. While the majority of studies assessing the impact of aerobic exercise on cardiopulmonary fitness have demonstrated an improvement in oxygen consumption [15–21, 23, 24], these studies have a number of limitations. All but one [19] had a small sample size, with 26 or less patients being exercised. Only three studies were randomised [18–20], only one prospectively assessed postoperative complications in a systematic way [19] and only two based their exercise program around the principles of HIIT [15, 18]. In this context there continues to be a need for well-designed randomised studies to confirm the extent of the impact of preoperative HIIT on oxygen consumption and to further assess what an optimal preoperative program of HIIT would look like [11].

Although we know that fitter patients do better [4–7], and there is increasing evidence that we can improve preoperative oxygen consumption, we have not yet been able to consistently demonstrate that this improvement in preoperative cardiopulmonary fitness will result in a subsequent reduction in postoperative complications [25]. Although in some reviews this has been assumed to be the case, and the evidence for this is beginning to accumulate, this assumption needs to be properly tested. Initial evidence supporting this assumption included the ability for prehabilitation to reduce the risk of some complications such as chest infection [13] and some studies demonstrating a reduction in hospital stay [13, 26]. This evidence has now been strengthened by two recent studies, published after our study commenced, which were designed to look at clinical endpoints. One, a study on abdominal aortic aneurysms, demonstrated a significant reduction in a composite endpoint including cardiac, respiratory and renal complications [19]. The second, a study of patients undergoing major abdominal surgery, demonstrated that the number of patients experiencing any complication halved from 62% to 31% [27]. However, at the time of designing our RCT, most studies did not show a reduction in length of stay and very few studies had systematically examined postoperative complications. In the context of most centres not being able to perform a study large enough to use complications as the primary outcome, we were interested in assessing what the impact of improving oxygen consumption would be, not only on postoperative complications, but also on a range of other relevant clinical outcomes. These included hospital stay, recovery from surgery and quality of life. We also wanted to assess whether the impact would be the same for patients across a range of pathologies and surgical procedures.

We have therefore designed a prospective RCT comparing an exercise program using HIIT against standard preoperative care for patients undergoing major abdominal surgery. The overall objective of this study is to examine the impact of a focused individualised HIIT exercise program over a 4–6-week period on cardiopulmonary fitness and postoperative complications. The hypothesis that we will be testing is that an individualised and supervised preoperative HIIT program will result in a clinically significant increase in Peak VO_2_ of 2 mL/kg/min among those patients who adhere to the program. A second hypothesis, which we will gather information to help assess in future studies, is that a clinically significant increase in Peak VO_2_ will result in clinically relevant improvements in length of hospital stay, complications and time to return to normal level of functioning after surgery.

The aims of the current study are to:

1. assess if an individualised and supervised preoperative HIIT program will result in a clinically significant increase in Peak VO_2_ of 2 mL/kg/min;
2. better understand the practical issues of performing HIIT in the cohort of patients requiring major abdominal surgery, which will include frail elderly people with significant co-morbidities;
3. examine the optimal individualization and delivery of HIIT to patients who are scheduled for major abdominal surgery; and
4. assess the feasibility of performing a larger clinical outcomes study looking at the impact of preoperative HIIT. To do this, we will explore the best endpoint for a subsequent RCT by examining a range of clinical endpoints in our current study. The results of the study will help us to decide what will be the most appropriate endpoint for a subsequent study; the distribution of measurements for this endpoint will enable us to calculate the number of patients required to perform an appropriately designed and powered study. This will also help us to determine if a multi-centre study will be required.

## Methods/design

### Study design

We are performing a single-centre, two-arm, parallel, prospective RCT comparing an exercise program using HIIT against standard preoperative care for patients undergoing major abdominal surgery.

### Recruitment to the study

The study is being performed at Dunedin Public Hospital, which is a tertiary care university hospital servicing a large geographical area in the south of New Zealand.

All patients we identify as fulfilling the inclusion criteria are being invited to participate in the study.

The inclusion criteria are:

1. patient requiring major abdominal surgery;
2. aged 45–85 years;
3. able to attend multiple supervised exercise sessions at the hospital;
4. available during a 4–6-week window up to the time of the confirmed surgery.

The definition of major abdominal surgery is any abdominal procedure expected to last 2 h or with an anticipated blood loss > 500 mL [28]. This included all gastrointestinal resections, liver surgery, large abdominal wall hernia repairs, abdominal aortic aneurysm repair, hysterectomy, radical prostatectomy and nephrectomy. For the purposes of this study, procedures such as laparoscopic cholecystectomy or transurethral resection of the prostate are not considered to be major abdominal surgery.

The exclusion criteria are related to medical risk factors and to conditions or treatments which could independently alter the Peak VO_2_ in the weeks before surgery. These include:

1. inability to exercise or to perform a CPET;
2. contraindication to exercise found on CPET;
3. uncontrolled hypertension (blood pressure > 180/100);
4. experiencing clinical angina (this does not include patients with a history of ischaemic heart disease who have no symptomatic angina on appropriate medical treatment or those who have previously had a successful revascularisation procedure);
5. myocardial infarction in the past three months;
6. uncontrolled cardiac arrhythmias;
7. aortic aneurysm > 6.5 cm;
8. severe obstructive pulmonary disease with a FEV1 < 1.0 L;
9. inability to provide consent;
10. significant anaemia defined as a haemoglobin level < 80 g/L or being on a treatment which would increase the haemoglobin in the 4–6 weeks before surgery;
11. preoperative chemotherapy during the 4–6 weeks before surgery; or
12. short course preoperative radiotherapy.

Protocol amendment number 01 was introduced in 19 April 2016 and increased the acceptable range for the age and the blood pressure of participants in the study to the levels listed above.

### Participant progress through the study

**Fig. 1:**
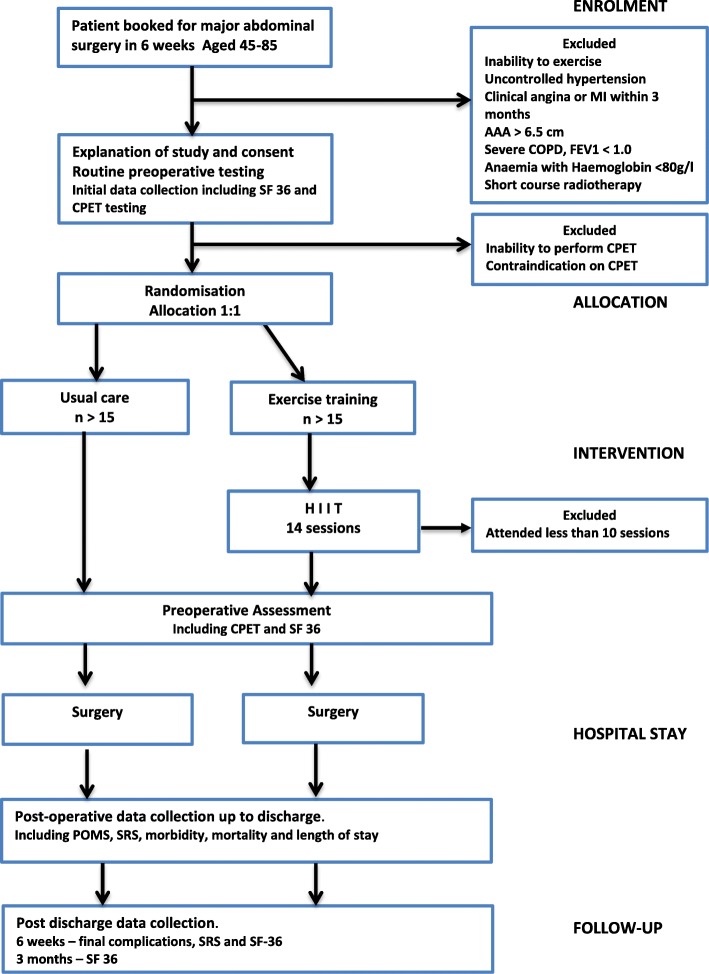
Study design

The study design is outlined in Fig. 1. In terms of patient care, all eligible participants have been assessed by their surgical team and by the hospital’s anaesthetic preassessment clinic. Potential participants for the study are identified when they are booked onto a list (with a specific date) for their operation. Those who are 4–6 weeks out from surgery, who live in or around Dunedin, and do not have any obvious exclusion criteria are contacted by the research nurse. Those who are interested are then invited to visit the hospital. Following discussion about the aims of the study and familiarisation with the study protocol, the participant is given written information about the study, is interviewed by the research nurse and is assessed by the study’s anaesthetist. When all questions are answered, and if there are no exclusion criteria, consent is obtained by the research nurse and the first cardiopulmonary exercise test (CPET) is performed. Baseline clinical data obtained before the CPET test includes full clinical history and examination, investigations done as part of the participant’s preoperative workup, up-to-date vital signs and a SF-36 quality of life score. The results of the CPET test are checked by a cardiologist and following this the participant is accepted into the study and randomly allocated 1:1 to standard care or to the exercise training group by the exercise physiologist. Randomisation was performed in blocks of unequal length (equal probabilities of 2, 4 and 6 lengths), using computerised sequence numbers generated in Stata, with no stratification. The allocation code is kept in sealed opaque envelopes which are sequentially opened. The allocation sequence was generated by the study statistician who has no involvement in the enrolment, assignment or assessment of participants. The enrolment of patients is coordinated by the research nurse. The number of patients approached, those excluded and those who declined to be involved (with reasons given for this) is also being documented as it the participant’s preference for exercise training or normal care.

### Interventions

Cardiopulmonary exercise testing (Quark CPET; Cosmed, Rome, Italy. Integrated gas analysis – Quark B2, integrated ECG analysis Quark C12) is performed in all participants while pedalling a stationary cycle ergometer (Quark Ergoline) immediately before randomisation and again before surgery. Before each test the metabolic analysers are calibrated as per the manufacturer’s recommendations. The oxygen (O_2_) and carbon dioxide (CO_2_) analysers were calibrated using a two-point calibration room air and a standardised gas containing 15% O_2_ and 6% CO_2_. The gas turbine was calibrated using a 3-L syringe (known volume) as instructed by the manufacturer. If the calibration failed, it was repeated until successful (software autocorrects if values fall below 5% error) before beginning the test. The test is performed in the hospital by two clinical researchers. After 3-min resting (for resting spirometry and to let gas exchange variables stabilise), the test proceeds with 2-min stages against an incremental resistance until termination, followed by 5 min of active recovery. Initial workloads were set at 25–50 W and stages increased by 10–50 W based on the participant’s fitness level and heart rate response. Heart rate, 12-lead electrocardiogram, blood pressure, and rated perceived exertion (RPE) are monitored throughout the procedure. The test was terminated when the participant had made a maximal effort (had reached volitional exhaustion). Additional evidence to support a good quality test included a respiratory exchange ratio (RER) of ≥ 1.1 or a plateau in the VO_2_ with increasing workload. If a participant terminates the test without reaching an RER of ≥ 1.1 this will be noted in the results. Additional medical reasons for terminating the test are discussed under safety. Other measures documented during CPET include the anaerobic threshold, resting and maximal heart rate (HRmax), blood pressure, the maximal workload in watts and the workload required to elicit 60% and 90% HRmax.

Participants are randomised either to standard care or exercise. Standard perioperative care at our hospital includes an anaesthetic preassessment clinic, a visit to the hospital with an orientation to enhanced recovery after surgery (ERAS) targets, a tour of the wards, treatment of anaemia, advice on stopping smoking and encouragement to exercise. However, as our institution has no staff with dedicated time for ‘optimising patients’ preoperatively there is no ability to follow up on advice given, especially with respect to exercise. For those randomised to ‘exercise’, the protocol is for 14 sessions of HIIT over 4–6 weeks. The frequency of sessions will be adjusted according to the number of weeks available, so that participants complete three or four sessions a week with the aim of completing their HIIT sessions three days before their scheduled surgery. The HIIT program uses stationary cycling on an electronically braked cycle ergometer (Monark Ergomedic 828E) and is performed under the supervision of a trained exercise physiologist. Each session begins and ends with 5 min of cycling against a light load. The frame work of the protocol is to start with ten 1-min intervals of intense exercise with the goal of reaching heart rates > 90% of HRmax with alternating ‘rest’ intervals of lower intensity cycling lasting 1 min at heart rates of ≥ 60% maximum heart rate. The duration of intense exercise intervals will increase as the participant gains fitness, with an aim of participants achieving five 2-min intervals of high intensity work with each high intensity interval being followed by 1–2-min of lower intensity cycling. During HIIT participants will alter the intensity of their exercise by manually adjust the resistance on their ergometers. This is reduced during periods of lower intensity cycling and is increased during periods of intense exercise to help achieve the target heart rate of 90% HR max. HRmax is defined as 220-age. The training programs will be individualised to the target heart rate and the performance of the participant. In this context, the protocol allows for individualised adjustments to accommodate patients with multiple co-morbidities. In these participants, the period of intense exercise will proceed for a time period that is reasonably tolerated (anywhere between 15 s to 1 min), followed by a period of lower intensity cycling for approximately 1 min. We would aim for this sequence to be repeated ten times. The duration of the intervals of intense exercise is then increased as tolerated in subsequent exercise sessions. In contrast, participants who are physiologically fit may rapidly increase the duration of intense exercise up to 4-min intervals. The total intense interval duration will not exceed 10 min throughout the training period. The training sessions, including warm up and cool down, last approximately 30 min. The impact of associated co-morbidities on the individualisation of the HIIT program will be noted. For patients on beta blockers, the level of perceived exertion, using the 6–20 Borg scale [29], will be monitored as well as the pulse, with a perceived exertion score of 18 being used as a substitute of 90% of HRmax. For safety reasons, the intensity of exercise and intervals will be adjusted if the heart rate exceeds 95% of the maximum observed on the baseline CPET or if the perceived exertion on the Borg scale exceeds 18. The pulse, as an indicator of ‘high intensity’ targets being reached, will be monitored and recorded from downloadable Polar Heart Rate monitors to ensure exercise adherence. This will be monitored by the exercise physiologist and the information is used to help with adjusting subsequent exercise sessions. Any patient who develops an AE will be clinically assessed and will be reported to the safety monitoring committee. All AEs will be reported with the results of the study. Patients who have their surgery delayed will complete one additional HIIT session per week up until surgery to maintain fitness [30, 32]. Adherence will be measured by the number of HIIT sessions attended and the number of sessions where a target HR of > 90% was reached.

### Outcomes

The primary outcome of our study is the change in Peak VO_2_. The peak VO_2_ is defined as the mean of the highest two rates of oxygen consumption measured during CPET [31]. This is documented after randomisation and again before surgery in all participants. One reason for measuring Peak VO_2_ a second time in the control group is that it enables us to identify any improvement in performance at the second CPET associated with becoming more familiar with the cycle ergometer.

We selected a range of secondary outcomes to assess the potential impact HIIT may have on patient wellbeing, recovery from surgery and a range of clinical outcomes. Although the study is not powered for these endpoints, we hope to gain sufficient information about them to assist with the design of subsequent studies. The two traditional clinical outcome endpoints that we are assessing are the length of hospital stay and postoperative complications. The length of stay is the number of postoperative days stayed in hospital, with day 1 being the day of surgery. Postoperative complications, using standard definitions [32, 33], are documented in hospital and after discharge. Complications in hospital will be prospectively identified by visiting the patient in the ward and from the patient’s medical records. The patient will then be contacted by a member of the research team six weeks after surgery. A previously validated questionnaire [1] is used to identify complications that developed after discharge from hospital. We are also documenting three composite postoperative scores. The postoperative morbidity survey (POMS) assesses postoperative adverse events in nine different categories according to predefined criteria that are easy to check and to document [34]. This gives an excellent overview of postoperative morbidity [28] and may give a more comprehensive overview of postoperative difficulties than identifying specific postoperative complications. The questionnaire is performed on postoperative day 5, either by direct interview in the ward or by telephone if the patient has been discharged. In contrast, the surgical recovery scale (SRS) is designed to look specifically at recovery after surgery. This assesses 13 items including energy levels, feeling of fatigue and a number of practical physical activities, and correlates well with major complications and length of hospital stay [35]. The SRS will be performed on postoperative day 5 and will be repeated at six weeks as a postal questionnaire. Third, we are documenting the quality of life using the short form 36 health survey (SF-36). In the context of this study, the SF-36 has two purposes. The first is as a quality of life measurement tool to assess the impact of HIIT on the patients’ overall wellbeing. The second is to assess if there are any differences in physical function after surgery, which can be assessed by using the ten questions which make up the physical functioning component of the score. On the first occasion the SF-36 is completed it will be performed with the patient. On subsequent occasions it will be completed as a postal questionnaire.

### Data management

**Fig. 2:**
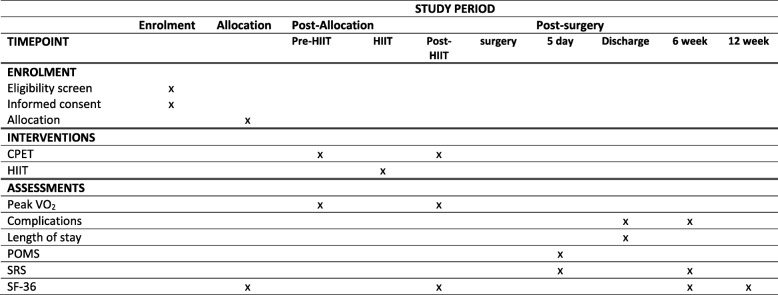
Schedule of enrolment, interventions and assessments

The schedule for data collection is summarised in the SPIRIT figure (Fig. 2) and checklist. The same dataset is collected for those randomised to exercise or to standard care. Each participant has a folder for demographic data, initial assessment and secondary endpoint results, and a separate folder for CPET results, details of the HIIT exercise program, including sessions attended and adherence to target heart rates. Folders are identified by the participant’s non-informative study number. Electronic data, for example for calculating SF-36 scores, will also be indexed by the participant’s study number and will be stored in a password-secured computer.

### Blinding

For the primary endpoint, Peak VO_2_, as the exercise physiologist supervises both the CPET test and the HIIT exercise program, he is not blinded. The Peak VO_2_ is measured at the point of maximal effort, which is reached with the encouragement of two independent researchers. In terms of secondary endpoints, the documentation of complications and administration of the questionnaires is performed by the principal investigator and a research assistant who are blinded to the group the patient has been randomised to. The study statistician will use non-informative study codes and remains blinded as to the actual allocation groups until all primary analyses are completed.

### Statistical analysis of sample size

As the literature, at the time of designing this study, did not provide the information needed to estimate the standard deviation for changes in Peak VO_2_ following 14 sessions of HIIT, it was decided to use an adaptive design, where the changes in peak VO_2_ in the exercise and control groups will be assessed after 30 patients have completed the study. At that stage, the required sample size to complete the study will be calculated using a group-blinded interim analysis where only the standard deviations will be calculated. No other statistical analyses will be performed before the conclusion of data collection. The only reason for stopping the study at this stage will be if sufficient participants have been recruited to meet the requirements of the sample size calculation.

### Statistical methods for analysis of endpoints

Patient demographics will be described using appropriate descriptive statistics (mean and standard deviation for normally distributed continuous variables, median and interquartile range for other continuous variables, count and percentage for categorical variables). The main statistical analyses will be directed at answering per-protocol questions around whether 14 (on a per-protocol basis we will accept ten or more) training sessions is efficacious at improving conditioning before surgery and at improving postoperative outcomes compared to no training sessions. Additional analyses will investigate all patients who were randomised in an intention-to-treat analysis (as allocated and using imputed data as needed). Differences in changes of Peak VO2 (the primary outcome) and SF-36 scores between the HIIT and usual care groups will each be separately modelled using linear regression with adjustment for baseline values (mixed for SF-36 with a random patient effect to accommodate the repeated outcome measurements at week 6 and month 3 and an interaction between group and time). The models will also adjust for age, surgery type and ASA score. Linear mixed models will similarly be used for the surgical recovery scores (day 5 and week 6) with a random patient effect, interaction between group and time, and adjusting for surgery type and ASA score. Linear regression will be used to compare length of stay (days) between groups adjusting for surgery type and ASA score. Usual model diagnostics will be used (including checking normality and homoscedasticity of residuals) and where necessary natural logarithmic transformation of dependent variables or quantile regression to model medians will be used. Differences between groups in postoperative complications (yes/no) and day 5 postoperative morbidity scale (0–9 and dichotomised) will be compared using Poisson regression with robust standard errors, providing estimated relative risks, adjusting for surgery type and ASA score. Missing data will be replaced using multiple imputation for both the per-protocol and intention-to-treat analyses with chained estimating equations. Statistical analyses will be performed using R 3.4.1 or Stata 15.0 and statistical significance will be determined by two-sided *p* < 0.05.

### Safety precautions and monitoring

Myocardial infarction continues to be one of the leading causes of unexpected postoperative mortality and is the main safety concern in this study. We are taking a cautious approach which includes steps to minimise cardiac risk at three stages throughout the study: during enrolment; before HIIT; and during HIIT. During enrolment, patients with established risk factors for a myocardial event are excluded. Before HIIT, performing the CPET to Peak VO_2_ is very similar to performing an exercise tolerance test, which is the gold standard for diagnosing unstable angina. Medical reasons for terminating the CPET include: exhaustion; extreme dyspnoea; light-headedness; chest pain; changes in the electrocardiogram tracing suggestive of ischaemia; complex cardiac arrhythmias; second- or third-degree heart block; a decrease in systolic pressure of 20 mmHg below the resting blood pressure; uncontrolled hypertension (systolic blood pressure of > 250 mmHg or a diastolic blood pressure > 120 mmHg); oxygen desaturation to < 80%; and signs of mental confusion. The guidelines from the American Heart Association confirm that, when following these guidelines, AEs are rare during properly supervised tests [36]. If a new diagnosis of a clinically important problem, such as ischaemic heart disease or a significant arrhythmia is made on CPET testing, the participant will not proceed to randomisation. The new diagnosis will enable appropriate preoperative management to be put in place, which would be expected to reduce the participant’s perioperative risk of complications. This happened in one of the first 20 participants. We believe that performing CPET up to Peak VO_2_, rather than to anaerobic threshold, increases the chance of identifying asymptomatic ischaemic heart disease during CPET, which will help to prevent unexpected problems during the exercise program. In terms of safety during the HIIT program, this is performed by an exercise physiologist, in the school of physical education. Safety criteria include a supervised and graduated workload, which is tailored to the target heart rate, age and co-morbidities of the individual. Hemodynamic and symptom monitoring is performed during and after exercise. An automated external defibrillator is present on site and there is a phone line to a designated arrest team in case of any cardiac event.

An independent safety monitoring committee of three medical consultants is in place. The committee has expertise in the areas of anaesthesia, surgery and cardiology. The role of the committee is to review the study protocols and to independently review any AEs related to the study interventions. The committee’s recommendations are binding and are directly reported to the principal investigator. Any patient harm caused by the study will also be eligible for compensation from the New Zealand Accident Compensation Corporation and will be reported with the study results.

### Ethics approval

The study underwent peer review within the University of Otago. Ethics approval was granted by the New Zealand Southern Health and Disability Ethics Committee, study reference number 15/STH/116. The study is registered with the Australian New Zealand Clinical Trials Registry (ACTRN12617000587303). The Universal trial number is U1111–1195-0805.

### Dissemination of results

The results of the trial will be communicated through publication in peer-reviewed journals and presentations in international conferences. A summary of the results will also be sent to the trail sponsors and will be made available to all those who participated in the study.

## Discussion

### Challenges in running the trial

This protocol paper provides us with an opportunity to share some of the challenges we have faced in running this trial. The greatest challenge has been with the recruitment of patients to the study, which has been contributed to by the location of, and organisational factors within, the hospital. With respect to our location, over half of eligible patients having major abdominal surgery live in rural areas too far away from the hospital to be able to attend regular exercise sessions. As we are performing a supervised training program, this places a major ‘distance’ limitation on recruitment. If this study shows that a preoperative HIIT program benefits the participants, a subsequent research question will be how this can be effectively rolled out to enable patients in distant communities to participate. With respect to the organisation of the hospital, for a complexity of reasons it is commonplace for lists not to be booked 4–6 weeks out from surgery. This has made coordinating the recruitment of patients difficult, with appropriate patients often ‘missing out’ on the opportunity to be involved. Different strategies to identify patients likely to have surgery, such as working with consultants and surgical secretaries have been tried, but with variable levels of success. Other reasons for difficulties with recruitment have included patients visiting relatives before their surgery, being busy at work and not being interested in participating in an exercise program. However, in terms of recruitment, one advantage of performing an exercise study in Dunedin is the compactness of the city with ‘universal’ easy access to the hospital. This may be one reason why we have been able to enrol patients with major co-morbidities who have been representative of the overall population of patients being booked for abdominal surgery.

We have also experienced problems with our randomisation to ‘standard care’. A number of the enrolled participants had been inactive and/or had significant health problems. The news of their surgery, and the opportunity created by the study, were motivating factors which contributed to making a conscious decision to improve their level of fitness. In this context, being randomised to standard care has often been a disappointment. Some participants, having made a decision to improve their level of fitness, have proceeded to organise their own exercise programs. Examples include joining a gym and a vigorous daily walk up a steep hill (of which there are many around Dunedin). One implication of this is that our ‘standard care group’ may no longer be an ideal ‘control group’. Because of this, we have asked participants in the control group to formally report back on the amount of exercise they are doing. We plan to exclude participants reporting substantial amounts of higher-intensity exercise (e.g. HIIT, hill walking, running) but not those reporting lower-intensity exercise (e.g., tai chi, walking on the flat, weight training) from the main per-protocol analysis. All patients recruited will be included in the intention-to-treat analysis. The other response to the disappointment of being randomised to ‘standard care’ has been for the participant to withdraw from the study as there is ‘no point in continuing’. So far this has happened twice. The disappointment with being randomised to standard care is therefore an important factor to consider when designing and interpreting RCTs in the area of prehabilitation. If we were designing the study again, we would we would make two changes. First, we would want to randomise between HIIT and an alternative intervention such as tai chi, strength exercises, respiratory exercises or dietary advice. Second, we would ask all participants to keep an exercise diary and to use a pedometer to help us accurately quantify the exercise undertaken by all participants.

In terms of design of the study and statistical analysis, issues with respect to insufficient data to perform a sample size calculation were discussed under ‘statistical analysis of sample size’. A second issue is the potential impact of chance differences in operations between the two groups on our secondary endpoints. Previous studies reporting on the perioperative use of aerobic exercise have usually been for patients having the same operation (such as a liver resection [18], anterior resection [15] or aortic aneurysm repair [19]). This present study includes patients having a range of different abdominal operations, which are known to have different rates of postoperative complications. Although differences in operations are not expected to impact on our primary endpoint (changes in preoperative Peak VO_2_), these may impact on our secondary endpoints. We will therefore adjust for surgery type and ASA score when performing our statistical analysis. By reducing the unexplained variance in the data, this should improve the precision of our estimates of intervention effects while also correcting for imbalances that arise by chance.

## Trial status

Enrolment in the trial started in September 2015. Initially the study was planned to finish in December 2017, but due to difficulties with recruitment, we have recently obtained an extension of funding to enable enrolment into the study to continue into 2018.

## Abbreviations

ASA: American Society of Anaesthesiologists physical status classification score
AT: Anaerobic threshold
CPET: Cardiopulmonary exercise testing
FEV1: Forced expiratory volume in one second
HIIT: High intensity interval training
HRmax: Maximal heart rate
Peak VO_2_: Peak oxygen consumption
POMS: Postoperative morbidity survey
RCT: Randomised controlled trial
RER: Respiratory exchange ration
SF-36: Short form 36 quality of life health survey
SRS: Surgical recovery scale
